# A Systematic Review of How To Reduce Morbidity in HIV Patients With Cardiovascular Diseases

**DOI:** 10.7759/cureus.34745

**Published:** 2023-02-07

**Authors:** Namratha Pallipamu, Sogand Taheri, Suvedha S Thiagaraj, Twisha S Shukla, Sai Dheeraj Gutlapalli, Hadi Farhat, Huma Irfan, Kanmani Muthiah, Michael Alfonso

**Affiliations:** 1 Medical Science, California Institute of Behavioral Neurosciences and Psychology, Fairfield, USA; 2 Internal Medicine, California Institute of Behavioral Neurosciences and Psychology, Fairfield, USA; 3 Pediatrics, California Institute of Behavioral Neurosciences and Psychology, Fairfield, USA; 4 Research, California Institute of Behavioral Neurosciences and Psychology, Fairfield, USA; 5 Internal Medicine, University of Balamand, Beirut, LBN; 6 Neurology, California Institute of Behavioral Neurosciences and Psychology, Fairfield, USA; 7 Medicine, California Institute of Behavioral Neurosciences and Psychology, Fairfield, USA

**Keywords:** highly active antiretroviral therapy, endothelial dysfunction, coronary artery diseases, cardiovascular diseases, antiretroviral therapy, human immunodeficiency virus

## Abstract

The human immunodeficiency virus (HIV) is known to cause cardiovascular diseases in patients infected with HIV. The pathology ranges from atherosclerosis to cardiomyopathy. There are several factors that could possibly cause cardiovascular diseases in the HIV population, including malnutrition and vitamin deficiency (for example, thiamine, B12, and zinc deficiencies); a lifestyle including increased prevalence of alcoholism and illicit drug usage; viral infection; and medication combinations that could cause sudden cardiac deaths. Cardiovascular diseases contribute to major morbidity in these populations and could have a reflection on the global financial burden, thus emphasizing the importance of prevention strategies. In this article, we focused on several factors that contribute to coronary artery disease and other cardiovascular diseases. We found that HIV has direct and indirect effects on the development of coronary artery diseases; furthermore, antiretroviral therapy adds to the deleterious effects of HIV and increases the risk of cardiovascular diseases. We further assessed the causal relationships and associations to understand the research gaps.

In conclusion, this paper acknowledges and summarizes the current management strategies and the need to develop future strategies focusing on the prevention of cardiovascular diseases and tailoring the regimens according to the patient’s clinical and socio-economic background.

## Introduction and background

Mankind has been battling the human immunodeficiency virus (HIV) for decades. It is understood that clinical cardiovascular diseases (CVD) tend to occur ten years ahead in HIV-infected populations than others [1]. In the 1980s, the most frequent cardiovascular complications in people living with HIV in developed countries were pericarditis and myocarditis caused by opportunistic infections, dilated cardiomyopathy, pericardial effusion, pulmonary hypertension, and cardiac tumors [1]. Over the past few decades, with the advent and usage of antiretroviral therapy (ART), opportunistic infections are kept in check and life expectancy is increased; however, people living with HIV (PLWH) are now presenting with an increase in mortality and morbidity due to coronary artery disease (CAD), atherosclerosis, and arrhythmias [1]. PLWH have a higher tendency to have traditional risk factors like hypertension, diabetes, and dyslipidemia. In addition, HIV infection causes dyslipidemia and chronic inflammation, leading to atherosclerosis triggers and progression [2]. Atherosclerosis is characterized by lipid storage and chronic inflammation in the arterial wall [3]. At the molecular level, reactive oxygen species and oxidative stress (OS) play a significant role in atherosclerosis. Studies showed that HIV infection, as well as ART, are associated with the development of oxidative stress, which mediates endothelial dysfunction [1,3]. Furthermore, the oxidative stress induced by HIV infection leads to apoptosis and causes plaque instability. Changes in lipid metabolism and hypercholesterolemia are associated with atherosclerosis. ART therapy, though it has shown significant efficacy in the suppression of the viral load, is associated with dyslipidemia and metabolic disorders like insulin resistance, diabetes mellitus, and lipodystrophy syndrome [3], which further increase the risk of cardiovascular disease in the HIV-infected population. Additionally, certain combinations of drugs in a highly active antiretroviral therapy (HAART) regimen could potentially increase the vulnerability to developing cardiovascular disease in HIV patients. For instance, ritonavir, which is known to cause dyslipidemia, when combined with lopinavir, increases the CVD risk, thus showing the ill effects of the ART regimen in PLWH. This article focuses on reviewing how far ART has helped us in the past few decades and how to improve the cardiovascular and overall health of the HIV-infected population.

## Review

Methods

We followed the Preferred Reporting Items for Systematic Reviews and Meta-Analyses (PRISMA) guidelines in our systematic review. The databases used were PubMed, PubMed Central, Medical Literature Analysis and Retrieval System Online (MEDLINE), and Google Scholar. Articles published in English from 2017 to 2022 were included in this systematic review. We obtained the information with the help of mesh terms and the following keywords: "human immunodeficiency virus", "antiretroviral therapy," "cardiovascular diseases," "coronary artery diseases," and "endothelial dysfunction" separately and in combination, and developed the following mesh strategy: (Human immunodeficiency OR Acquired immunodeficiency syndrome OR "HIV/drug effects"(Majr) AND Antiretroviral therapy OR ("Antiretroviral Therapy, Highly Active/adverse effects"(Majr) OR "Antiretroviral Therapy, Highly Active/mortality"(Majr)) AND cardiovascular diseases OR coronary artery diseases OR endothelial dysfunction OR ("Cardiovascular Diseases/mortality"(Majr) OR "Cardiovascular Diseases/physiopathology"(Majr)) to find the relevant information.

**Figure 1:**
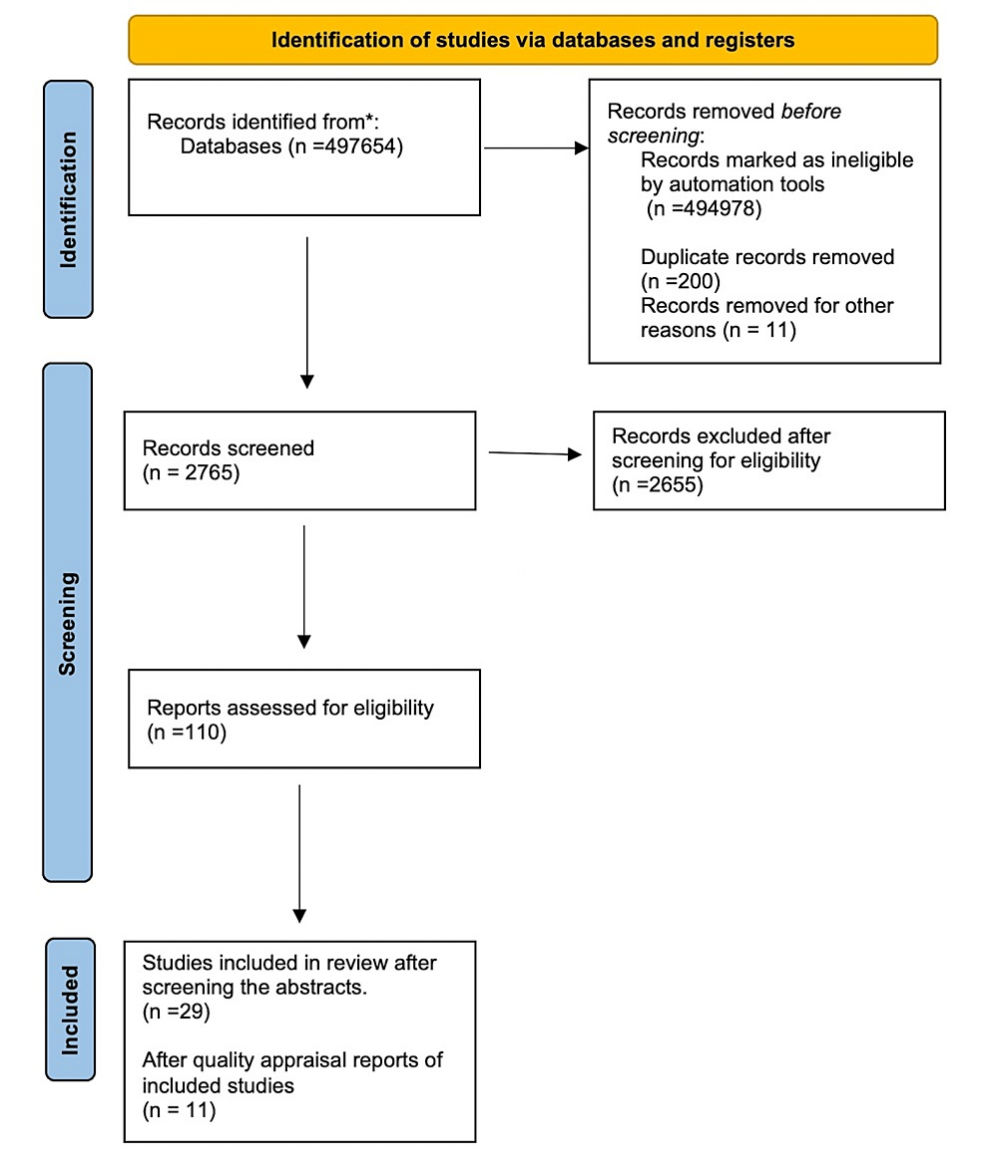
PRISMA flow diagram PRISMA: Preferred Reporting Items for Systematic reviews and Meta-Analyses

Our data search turned up 497,654 results. We identified and eliminated duplicates, then screened 2765 articles by title and abstract and collected 110 articles. We then applied eligibility criteria and quality assessment tools to 29 relevant articles; we included 11 of the finally selected articles after the quality assessment in this study. Figure 1 below shows the PRISMA flow diagram.

Data extraction and quality appraisal: The quality of all 11 studies was assessed based on different tools for each type of study. a) Cochrane risk bias assessment tool for randomized clinical trials; b) Newcastle-Ottawa scale for observational studies; c) Measurement Tool to Assess Systematic Reviews (AMSTAR2) was used to assess the quality of systematic reviews; d) Scale for the Assessment of Non-Systematic Review Articles (SANRA) checklist to assess the quality of reviews.

Inclusion and Exclusion Criteria

People living with HIV who were taking antiretroviral therapy and who developed cardiovascular diseases along the course of the disease were included in articles between 2017 and 2022 in English-language literature, clinical studies, review studies, observational studies, and cross-sectional studies to derive relevant information for this systematic review. Articles in languages other than English, letters to the editor, books, and articles older than five years were excluded.

Results

Of all the relevant articles found, 11 have been analyzed, and the results are presented below. These studies helped to evaluate the direct effects of HIV and ART and also to analyze and weigh the risks and benefits of antiretroviral therapy in patients with HIV. Table 1 below shows the data extraction table that was constructed based on the data from the finally selected articles.

**Table 1:** Data extraction table ART: antiretroviral therapy; OS: oxidative stress; HIV: human immunodeficiency virus; AIDS: acquired immunodeficiency syndrome; HAART: highly active antiretroviral therapy; CVD: cardiovascular diseases; PLWH: people living with HIV; PLWHA: people living with HIV/AIDS; SCD: sudden cardiac death; ASCVD: atherosclerotic cardiovascular disease

S.No	Author	Year of Publication	Purpose of study	Type of Study	Sample size	Conclusion and Results
1	Akkoyunlu Y [4].	2020	To investigate the effect of integrase inhibitor-based antiretroviral therapy on oxidative stress parameters and thiol-disulfide homeostasis in people living with HIV.	A prospective cohort study	30	The study concluded that integrase inhibitor-based ART decreases the OS caused by HIV infection. As PLWH needs lifelong treatment, integrase inhibitor-based ART may be a good therapeutic option.
2	Emad Mogadam [5].	2020	This study intended to investigate indicators of endothelial dysfunction without traditional risk factors (hypertension and diabetes) in the HIV cohort.	A retrospective cohort study	19	It was concluded that a lower nadir cluster of differentiation 4 (CD4) T cell count nadir was independently associated with worse endothelial function in HIV-infected patients, despite effectively suppressed viral load and without hypertension, diabetes, or clinical evidence of CVD.
3	Erildo Vicente Muller [6].	2019	This study aimed to describe metabolic changes in HIV/AIDS patients according to their treatment regimen.	A retrospective cohort study	538	When evaluating the association between HIV/AIDS and exposure to HAART, it was concluded that CVD was prevalent in PLWH taking the HAART regimen. It was concluded that HIV/AIDS risk factors and other differentiated measures should be considered in the care of these patients, including consideration of the clinical and sociodemographic profiles of patients, to implement early prevention strategies.
4	Fahim Pyarali [7].	2021	The goal of this study is to identify the prevalence of CVDs in PLWH in south Florida and assess the use of aspirin and statins for primary and secondary prevention of CVDs in those populations.	Retrospective chart review	985	This study concluded that, in light of the increased risk of CVD in PLWH on ART, there is a need to implement interventions to increase awareness of CVD risk reduction strategies among PLWH providers. In addition, this study emphasized the need for strategies to overcome the barriers to effective primary and secondary prevention of CVD after exploring the beliefs regarding CVD prevention to reduce CVD mortality and morbidity.
5	Yung-Feng Yen [8].	2019	This study aimed to determine sudden cardiac death (SCD) risks in Taiwanese patients with and without HIV infection.	A prospective cohort study	24,306 PLWH and 97,224 (matched controls)	Concluded that HIV is an independent risk factor for SCD and that PLWH on ART have a lower incidence of SCD, as it significantly controls the viral load and thus endothelial dysfunction and atherosclerosis, and also lowers the incidence of cardiomyopathy.
6	Yung-Feng [9].	2019	This study is aimed at examining the association of HIV infection and HAART with the risk of incident heart failure during 2003–2014 in Taiwan.	Retrospective cohort study	120,765 patients (24,153 PLWHA and 96,612 matched controls)	HIV is an independent risk factor for incident heart failure (HF). The incidence rate of heart failure (HF) per 100,000 person-years was 136.34 in the PLWHA and 79.54 in the control group (P < 0.001). Based on univariate analysis, in PLWHA receiving HAART vs. not receiving HAART, the rate of incidence was 163.01 and 108.62 per 100,000 person-years, respectively (P < 0.001). When the incidence of HF was assessed based on the duration of exposure to HAART, it was observed that the incidence of HF decreased as the duration of exposure to HAART increased (P = 0.014).
7	Paschal O Njoku [10].	2021	The objective of this study was to evaluate the clinical and echocardiographic findings in HIV-infected adults.b	Cross-sectional study	One hundred HIV subjects on HAART, 100 HAART-naïve patients, and 100 controls	Dimensions of the aortic root (2.71 cm), left atrium (3.27 cm), and left ventricular mass index (79.95) were relatively higher in HIV-positive subjects on HAART (p < 0.05). LVEF and wall thickness are higher in the HIV-positive, HAART-naive group. However, clinical features of HIV infection, such as immunosuppression and a reduced CD4 cell count, were more prevalent in HIV-positive patients who were HAART-naive than in HIV-positive patients on HAART.
8	Ajala Aisha Oluwabunmi [11].	2019	The goal of the study is to evaluate the risk of cardiovascular disease (CVD) in patients taking HAART.	Cross-sectional study	100 HAART-experienced HIV seropositive persons and 100 age- and sex-matched, seropositive, but HAART-naive controls	This study has shown that people living with HIV on HAART have a higher prevalence of cardiovascular risk factors (hypertension, dyslipidemia, and overweight or obesity) when compared to age- and sex-matched controls who are HIV positive but HAART-naïve. These risk factors contribute in a multiplicative manner to increase their overall risk for CVD.
9	Sakaewan Ounjaijean [12].	2021	This research aims to explore the importance of protease inhibitors (PI) in CAD pathogenesis among Asian populations.	Cross-sectional study	boosted-PI group, N = 30 NNRTI-based ART (non-PI group, N = 30)	Compared with non-PI, patients in the boosted-PI group had more evidence of dyslipidemia. Circulating levels of inflammatory markers, C-reactive protein (CRP) (5.4±9.1vs. 14.9 ± 19.4 mg/L,p= 0.019), and lectin-liked oxidized lipoprotein receptor-1 (LOX-1), (387 ±299 vs.554 ± 324 pg/mL, p = 0.042), were lower in the boosted-PI group. Contrastingly, vascular adhesion molecules-1 (VCAM-1) (160.2 ± 80.0 vs. 147.8 ± 66.3 ng/mL, p = 0.010) and osteoprotegerin (OPG) (153.7 ± 57.1 vs. 126.4 ± 35.8, p = 0.031) were higher. After adjustment in the multivariate analysis, PI treatment is the only independent parameter associated with the changes in CRP, LOX-1, VCAM-1, and OPG.
10	Tecla M. Temu [13].	2020	The aim of the study was to examine the relationship between monocyte activation and endothelial activation in PLWH in Kenya.	Cross-sectional study	541 adults: 275 PWH on ART and 266 HIV-negative adults	This study concluded that monocyte activation may explain the increased risk of endothelial dysfunction and CVD, independent of traditional risk factors.
11	Jaruwan Tiarukkitsagul, [14].	2021	This study is aimed at assessing the 10-year ASCVD risk of PLWH receiving first-and second-line ART.	Cross-sectional study	460	The prevalence of 10-year ASCVD is high in PLWH taking ART; especially, the prevalence is higher in patients taking second-line ART, and second-line ARTs were found to be an independent predictor of high 10-year ASCVD risk. So in PLWH, and especially in those who are taking second-line ART, the ASCVD risk should be assessed.

Discussion

Humanity has been dealing with the human immunodeficiency virus (HIV) for the past several decades. In the era of antiretroviral therapy (ART), people living with HIV (PLWH) have seen a drastic improvement in the disease course, quality of life, and life expectancy. Although increased life expectancy is a reflection of the success of ART, people living with HIV (PLWH) are at increased risk of cardiovascular diseases, including myocardial infarction (MI), heart failure (HF), and pulmonary hypertension (PH) [15], which stand on the top list of morbidity and mortality among PLWH, while CVDs in addition to diseases of aging in this population increase the burden on health care globally [16]. Studies have shown that PLWH remains at high risk of heart failure (HF) and subclinical systolic and diastolic dysfunction. A sustained increase in the risk of heart failure in this population can be attributed to other factors like higher rates of traditional risk factors for CHF, CAD, drug abuse, and PLWH, who are also known to have higher levels of autonomic dysfunction and chronic inflammation, which are novel risk factors for heart failure in the general population [17]. In a study conducted by Yen YF, Ko MC, Yen MY, et al., HIV was found to be an independent risk factor for heart failure [9]. Some ARTs, like zidovudine, cause mitochondrial damage, causing myocardial necrosis and increasing the risk of heart failure [10]. Pulmonary arterial hypertension (PAH) is another under-recognized presentation in PLWH, resulting in right ventricular failure and death, but studies showed a statistically significant difference in the degree of PAH in PLWH vs. the HIV-uninfected population. The prevalence (0.5%) of HIV-associated PAH has not changed with the use of ART. In addition, there was no correlation between HIV-associated PAH and the cluster of differentiation 4 (CD4+) cell count. Considering this, PAH may remain undiagnosed until the late stages of PLWH. Therefore, further efforts to implement screening guidelines for these patients would benefit their outcomes [17]. As mentioned earlier, CAD is one of the top five causes of death in PLWH, so understanding why PLWH have accelerated atherosclerosis and related pathologies is needed in order to provide appropriate care and treatment for this population [18,19]. It is estimated that several factors contribute to cardiovascular mortality in PLWH. One of them is the increased life expectancy in PLWH and the age-related cardiovascular changes; others include HIV infection, poor infection control, chronic inflammation, immunologic progression (low CD4+ cell count), and ART-mediated cardiometabolic dysfunction [7, 15, 19].

HIV Infection and Its Contribution

HIV infection or AIDS has a high prevalence of hypertension and diabetes [5] and is associated with high lipid disorders as plasma viremia promotes dyslipidemia-a decrease in the total cholesterol (TC), high-density lipoprotein (HDL), and low-density lipoprotein (LDL)-and an elevation in the concentration of triglycerides (TG) in the later stages. The reduction in HDL that occurs due to activation of the immune system in early HIV-1 infection promotes lipid peroxidation and inflammatory cytokine production, thus promoting an imbalance in the antioxidant system, which increases the chance of atherosclerotic diseases [3]. In normal conditions, there is a balance between thiols, which are important antioxidants and contribute up to 52.9% of serum total antioxidant status (TAS), and disulfide (DIS). HIV infection is known to induce oxidative stress (OS) [20]. In addition, HIV generates reactive oxygen species (ROS) via the transactivator of transcription (TAT) protein and causes damage that is independent of CD4+ count, HIV viral load, and subclinical inflammation [4], thus questioning the benefits of keeping HIV viral load and CD4+ levels under control in regards to cardiovascular health. Also, in a study conducted by Mogadam E, King K, Shriner K, et al., it was shown that there was a strong independent association between a lower CD4+ T cell nadir and a low vascular reactivity index (VRI), indicating poor endothelial function, despite undetected viral loads and no traditional risk factors in the study participants. However, higher viral loads are associated with the worst endothelial dysfunction or damage, which is an important factor that contributes to atherosclerosis [5].

Furthermore, HIV seroconversion creates a continuous state of immune activation that leads to T-cell, monocyte, and macrophage-mediated inflammation, despite highly active antiretroviral therapy (HAART). This inflammatory condition interacts with the coagulation factors and leads to endothelial dysfunction, which increases the risk of cardiovascular events. Cardiomyopathy occurs as a result of the direct invasion of HIV onto the myocytes, which leads to lymphocytic infiltration and necrosis of the adjacent cells. This is associated with the progressive development of CHF and arrhythmia. Nonetheless, there is no benefit to adding an anti-inflammatory agent to HAART in HIV-infected patients in improving the CVD endpoints [1].

Anti-Retroviral Therapy

Treatment of HIV has been aimed at targeting different steps of the HIV life cycle. The therapy consists of two nucleoside reverse transcriptase inhibitors (NRTI) and one protease inhibitor (PI) or one non-nucleoside reverse transcriptase inhibitor (NNRTI) or one integrase inhibitor and a C-C chemokine receptor 5 (CCR5) inhibitor [21]. Previous research concluded that, besides their substantial benefits, ART is associated with metabolic changes and increased CVD risk, including atherosclerosis [3,6,7,16]. Few antiretroviral drugs are known to induce OS by several biochemical reactions. For example, nuclease reverse transcriptase inhibitors (NRTI) induce mitochondrial toxicity [11], and protease inhibitors (PI) activate hepatic cytochrome p450. In addition, protease inhibitors (PIs) are associated with dyslipidemia and fibrinogenemia, and non-nucleoside reverse transcriptase inhibitors (NNRTI) increase total cholesterol and LDL-C; efavirenz causes hyperlipidemia, reflecting the usage of highly active antiretroviral therapy (HAART) and its association with several damaging effects on the cardiovascular system leading to CVDs [11]. However, considering the benefits vs. risks, it was shown that the OS caused by ART is less when compared to the OS caused by HIV infection [4]. The Standardisation of Radiotherapy (START) trial also showed that HIV-positive (HIV+) individuals on ART have a more favorable CVD risk profile when compared to those not on ARTs. In a study by Y Akkoyunlu, it was observed that integrase inhibitor-based ART therapy decreases OS caused by HIV infection. In light of the need for lifelong ART in PLWH, an integrase-based regimen could be a better option [4]. Data from Muller EV and Gimeno SGA’s study showed that the prevalence of CVD is high in PLWH taking the HAART regimen, considering the metabolic changes observed in those populations [6]. Though the origin of these presentations is not fully understood, it is believed to implement early prevention and treatment strategies, tailored according to individual patients’ backgrounds. In a cross-sectional study conducted by Jaruwan Tiarukkitsagul and Somnuek Sungkanuparph, there was a significant 10-year risk of ASCVD among PLWH, especially among patients taking second-line ART, a ritonavir-boosted PI, in comparison to first-line ART, an NNRTI-based regimen. The second-line ART was found to be an independent risk factor for intermediate to high 10-year ASCVD risk [14].

Can We Improve Overall Health?

So far, it has been well established that CVD in PLWH is due to the intertwined effects of traditional risk factors, ART, and HIV infection-mediated effects. Despite the potentially harmful effects of ARTs on cardiovascular health, considering the benefits vs. risks, ARTs have brought substantial health improvement and increased the life expectancy of HIV/AIDS patients. In addition, considering alternate regimens like integrase inhibitor-based anti-retroviral therapies are advised as PLWH needs lifelong therapy [4]. Furthermore, it was found that PLWH on ART have a lower incidence of sudden cardiac deaths (SCDs) and cardiomyopathy [8]. It was also found that an increased duration of exposure to HAART decreases the incidence of heart failure [9]. Therefore, we need to focus on improving cardiovascular health by implementing screening and preventive strategies to reduce CVD mortality and morbidity [7].

Aspirin and Statins

Aspirin, or acetylsalicylic acid (ASA), is a non-steroidal anti-inflammatory drug (NSAID) with anti-inflammatory and anti-platelet properties. ASA has been found to have beneficial effects, including halting the disease progression in HAART-naive patients, increasing the CD4+ counts, decreasing the p24 antigen, and improving body weight and hemoglobin levels [22]. Considering the significant benefits of aspirin, the addition of this drug to ART is advised. However, literature shows that nonadherence and polypharmacy could be potential concerns [22,23]. Considering the side effects of aspirin in higher doses, including GI bleeding, the use of aspirin in higher doses can cause additional side effects like gastrointestinal (GI) bleeding, which would increase the discomfort in PLWH. Therefore, additional studies observing the benefits of adding low-dose aspirin to ART are needed to identify the true benefits of aspirin in PLWH. Statins are widely used in the primary and secondary prevention of CVD in the general population. Though they have the potential to prevent CVD in PLWH, they are being underprescribed by clinicians [16,24]. The reasons for this and their usage spread over a wide spectrum, ranging from clinician-level factors, including limited knowledge of prescribing guidelines, all the way to patient-related factors like non-adherence and a lack of knowledge regarding the benefits of statins [16]. In a randomized pilot trial conducted by Eugènia Negredo et al. to study the benefit of adding atorvastatin to raltegravir in PLWH, the addition of statin didn’t show a difference in the lipid profile, nor did it provide significant immunological benefits in terms of a reduction in inflammatory markers or immune activation markers [25]. Another major concern in the usage of statins in PLWH is their interaction with ART drugs [26]. The rationale and design of the Randomized Trial to Prevent Vascular Events (REPRIEVE) in the HIV trial in 2019 used pitavastatin, which minimally interacts with ART to prevent CVD. This trial found that the moderate-intensity regimen using pitavastatin 4 mg/day reduced low-density lipoprotein C (LDL-C) more effectively when compared to the 40 mg/day of pravastatin.

Limitations

We encountered a few difficulties in finding the articles pertinent to our question; initially, we tried to pull the articles that would focus on the age range between 19 and 44, but due to the limited availability of literature, we removed that criterion and focused on patients living with HIV aged more than 19. We couldn’t include literature from languages other than English. We considered papers from the past five years and didn’t focus on literature older than five years despite having useful information, and because of these filters, many valuable articles could have been overlooked.

## Conclusions

This paper summarizes the benefits and harmful effects of ART and emphasizes the importance of implementing preventive strategies in managing PLWH on ART. We focused on knowing the cardiovascular disease presentations in people living with HIV and how effective antiretroviral therapy is for those patients. From our research, it can be concluded that ART therapy has substantial benefits for PLWH. We need to keep in mind that ART could potentially have harmful effects on overall cardiovascular health; however, considering the overall benefits for a patient with HIV, including lowering the incidences of sudden cardiac deaths and heart failure when compared to patients not taking ART, it is considered effective in improving the morbidity of patients living with HIV. Therefore, we need to keep an emphasis on screening and preventive strategies and tailor the regimens according to the individual patients’ backgrounds to improve the overall health of this population. Further studies and strategies are needed to narrow down and show evidence of how certain strategies can benefit PLWH on the ART regimen.
